# FINDINGS FROM THE ENTOURAGE NOORD PROJECT: CO-CREATING NEW HOUSING MODELS FOR OLDER ADULTS IN BRUSSELS

**DOI:** 10.1093/geroni/igz038.941

**Published:** 2019-11-08

**Authors:** An-Sofie Smetcoren, Liesbeth De Donder

**Affiliations:** Vrije Universiteit Brussel, Brussels, Belgium

## Abstract

In response to the challenge of an ageing population and the housing crisis in Brussels, the practice-based project ‘Entour-Age Noord: Inspiring and innovative housing & work’ was launched. A main objective was to develop various innovative and small-scaled housing models for older adults. The new housing models were designed to reinforce quality of life of older people who are ageing in the neighbourhood whilst allowing them to choose the models best suited to their needs and wishes. Given the complexity and multidimensionality, participatory-action research was used and the project was divided into six different ‘work packages’ (WP’s). Within these WP’s, different stakeholders and end users (older people, informal caregivers, neighbourhood residents, Community Land Trust, etc.) were involved during various activities (service design methodology, architectural workshops, inspiring visits, focus groups etc.) in order to co-create the answers. First, based on the results, eight personas (=conceptual models of targeted groups) were developed. Second, stemming from the participants needs and wishes (e.g. desire for more social interaction), architectural and spatial design characteristics were detected (e.g. provide common/shared spaces that stimulate encounter). Based on these personas and spatial characteristics, two prototypes of new housing models were conceptually elaborated on paper and two were operationalized and prepared in practice. The spatial and architectural characteristics are not limited to old age but can be of interest of any age group. Future housing developments could take these suggestions into consideration. Furthermore, experiences, opportunities and pitfalls of a co-creative decision-making process with older adults will be discussed.

